# Phosphorylation of a WRKY Transcription Factor by MAPKs Is Required for Pollen Development and Function in *Arabidopsis*

**DOI:** 10.1371/journal.pgen.1004384

**Published:** 2014-05-15

**Authors:** Yuefeng Guan, Xiangzong Meng, Reshma Khanna, Erica LaMontagne, Yidong Liu, Shuqun Zhang

**Affiliations:** 1 Division of Biochemistry, Interdisciplinary Plant Group, and Bond Life Sciences Center, University of Missouri, Columbia, Missouri, United States of America; 2 Shanghai Center for Plant Stress Biology, Chinese Academy of Sciences, Shanghai, China; Leicester University, United States of America

## Abstract

Plant male gametogenesis involves complex and dynamic changes in gene expression. At present, little is known about the transcription factors involved in this process and how their activities are regulated. Here, we show that a pollen-specific transcription factor, WRKY34, and its close homolog, WRKY2, are required for male gametogenesis in *Arabidopsis thaliana*. When overexpressed using *LAT52*, a strong pollen-specific promoter, epitope-tagged WRKY34 is temporally phosphorylated by MPK3 and MPK6, two mitogen-activated protein kinases (MAPKs, or MPKs), at early stages in pollen development. During pollen maturation, WRKY34 is dephosphorylated and degraded. Native promoter-driven WRKY34-YFP fusion also follows the same expression pattern at the protein level. WRKY34 functions redundantly with WRKY2 in pollen development, germination, and pollen tube growth. Loss of MPK3/MPK6 phosphorylation sites in WRKY34 compromises the function of WRKY34 *in vivo*. Epistasis interaction analysis confirmed that *MPK6* belongs to the same genetic pathway of *WRKY34* and *WRKY2*. Our study demonstrates the importance of temporal post-translational regulation of WRKY transcription factors in the control of developmental phase transitions in plants.

## Author Summary

Pollen development, or male gametogenesis, is a process by which a haploid uninucleate microspore undergoes cell division and specification to form a mature pollen grain containing two sperm cells. The highly defined cell linage makes pollen development an ideal model to understand the regulation of plant cellular development. Pollen development has multiple phases and involves dynamic changes in gene expression, which highlights the importance of transcription factors and their regulatory pathway(s). In this report, we demonstrate that WRKY34 and WRKY2, two closely related WRKY transcription factors in *Arabidopsis*, play important roles in pollen development. WRKY34 is phosphorylated by MPK3/MPK6, two functionally redundant mitogen-activated protein kinases (MAPKs or MPKs), at early stages in pollen development. Utilizing a combination of genetic, biochemical, and cytological tools, we determined that this MAPK-WRKY signaling module functions at the early stage of pollen development. Loss of function of this pathway reduces pollen viability, and the surviving pollen has poor germination and reduced pollen tube growth, all of which reduce the transmission rate of the mutant pollen. This study discovers a novel stage-specific signaling pathway in pollen development.

## Introduction

Pollen, the male gametophyte of angiosperms, displays highly reduced structure of two or three cells at maturity. Because of the simple cell linage and dynamic developmental processes, plant male gametogenesis provides an interesting model for studying many fundamental cellular processes, including cell specification, cell polarity, cell cycle, and transcriptional regulation in these processes. During male gametogenesis, the uninucleate microspore (uninucleate microspore stage, UNM) undergoes an asymmetric mitosis to generate a large vegetative cell and a generative cell within it (bicellular pollen stage, BCP). In *Arabidopsis thaliana*, before pollen maturation, the generative cell undergoes a second symmetric mitosis to create two sperm cells (tricellular pollen stage, TCP). Prior to anther dehiscence and pollination, the TCP further develops into dehydrated mature pollen (mature pollen stage, MP) [1]. Pollen development is highly regulated, which is associated with successive global transcriptional regulation throughout the process [2], [3].

The precise and dynamic regulation of male gametogenesis requires transcription factors. In *Arabidopsis*, over 600 transcription factors are expressed during male gametogenesis, which forms a dynamic regulatory network [2], [4]. A subset of pollen-specific MIKC* MADS box proteins (AGL30/65/66/94/104) are expressed preferentially during pollen maturation [2], [5]. Double mutant combinations revealed the important roles these genes play in pollen germination and pollen fitness [5]. In *Petunia*, seven different zinc-finger transcription factors are expressed transiently and sequentially at different stages of pollen development [6]. Such transcription factors might each have specific target genes and constitute a regulatory cascade during pollen development [6]. Although progress has been made on the potential importance of transcription factors in male gametogenesis, little is yet known about the biological function of these transcription factors and how their activities are regulated to form temporal transcriptional regulatory networks.

Besides expression regulation, post-translational modification is a common mechanism to regulate the activity of transcription factors. Phosphorylation/dephosphorylation through mitogen-activated protein kinase (MAPK) cascades is a conserved post-translational modification in eukaryotes. A MAPK cascade minimally consists of three kinases: a MAPK, a MAPK kinase (MAPKK), and a MAPKK kinase (MAPKKK). The activity of MAPKs is regulated by their upstream MAPKKs through phosphorylation, and MAPKKs are activated through phosphorylation by their upstream MAPKKK(s). MAPKKKs are downstream of receptors/sensors and are activated in response to extracellular stimuli or to developmental signals [7]. Once activated, MAPKs can phosphorylate functionally divergent substrates on serine or threonine residues within a minimal S/T-P motif [8]. In *Arabidopsis*, there are 20 MAPKs, of which MPK3 (At3g45640) and MPK6 (At2g43790) are extensively studied. MPK3 and MPK6 have been revealed to phosphorylate multiple substrates, including transcription factors, in diverse biological processes [9]–[14]. For instance, WRKY33 (At2g38470) is a WRKY transcription factor required for pathogen defense in *Arabidopsis* [15]. In response to *Botrytis cinerea* infection, WRKY33 is phosphorylated by MPK3/MPK6, which is important for the activation of WRKY33, as mutations of MAPK-phosphorylation sites compromise the function of WRKY33 *in vivo* [9].

In this report, we show that WRKY34 (At4g26440), a new substrate of *Arabidopsis* MPK3/MPK6, is involved in male gametogenesis. WRKY34, a close homolog of WRKY33, is a pollen-specific WRKY transcription factor. When overexpressed using *LAT52*, a strong pollen-specific promoter, WRKY34 protein is temporally phosphorylated by MPK3/MPK6 at early stages in pollen development and then becomes dephosphorylated and degraded right before pollen maturation. Loss-of-function genetic analysis shows that WRKY34, together with a close homolog WRKY2 (At5g56270), plays important roles in pollen development and function. A complementation assay suggests that the phosphorylation of WRKY34 by MPK3/MPK6 is important for its function *in vivo*. Although single mutation of none of the *WRKY2*, *WRKY34*, *MPK6* genes causes a pollen developmental defect, both the *wrky2 mpk6* and *wrky34 mpk6* double mutants exhibit pollen developmental defects similar to the *wrky2 wrky34* double mutant, demonstrating that *WRKY34* and *WRKY2* indeed belong to the same pathway of *MPK3/MPK6* in early pollen development.

## Results

### WRKY34 is phosphorylated by MAPKs *in vitro*

**Figure 1:**
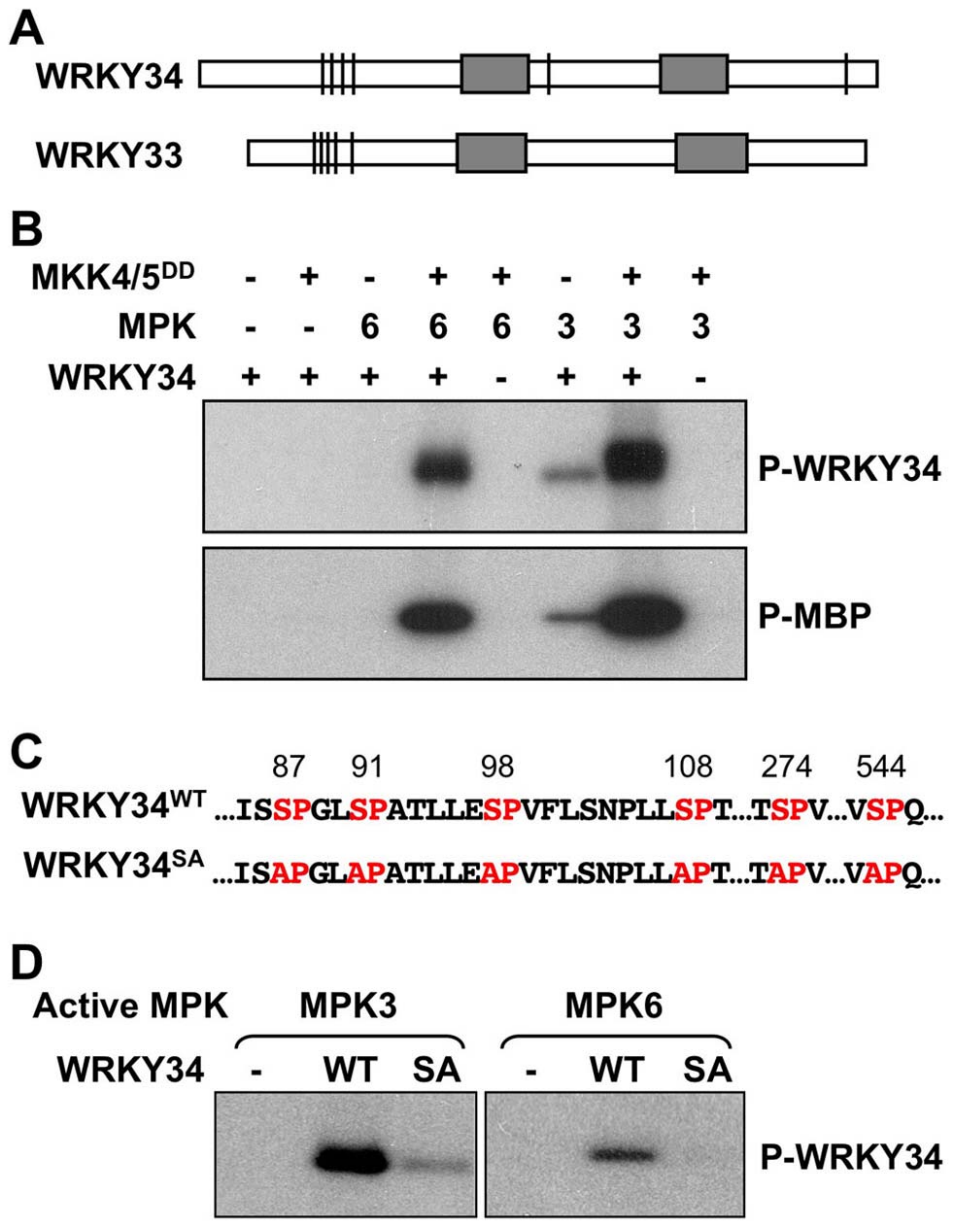
*In vitro* phosphorylation of WRKY34 by MPK3 and MPK6. (A) Putative MAPK-phosphorylation sites in WRKY34 and WRKY33. Bars indicate the position of potential MAPK phosphorylation sites in the protein. Grey boxes indicate WRKY domains. Note that the clusters of phosphorylation sites at N-termini were similar between WRKY34 and WRKY33. (B) *In vitro* phosphorylation assay of WRKY34 by the activated MPK3 and MPK6 (upper panel). Reactions with various components omitted (-) were used as controls. Recombinant MKK4^DD^/MKK5^DD^ were used to activate MPK3 and MPK6. Myelin basic protein (MBP) was used as control substrate (lower panel). (C) Adjacent sequences of putative MAPK-phosphorylation sites in WRKY34, and the loss-of phosphorylation WRKY34 mutant with all Ser mutated to Ala (WRKY34^SA^). (D) Mutation of MAPK-phosphorylation sites greatly reduced the phosphorylation of WRKY34 by MPK3 and MPK6. Phosphorylated WRKY34 was visualized by autoradiography after gel electrophoresis.

After the identification of WRKY33 as a substrate of MPK3/MPK6 in regulating plant defense responses [9], [16], we examined other WRKYs that share high homology with WRKY33 for potential MAPK phosphorylation sites. WRKY transcription factors are divided into three groups based on the number of WRKY domains (two copies in Group I, and one copy in Groups II and III) and the structure of their zinc fingers (C2HC in Group III but not in Group II proteins) [17]. WRKY33, with two WRKY domains, belongs to Group I in the WRKY family [17]. WRKY34 (At4g26440), another Group I member that shares high homoxlogy with WRKY33, is a pollen-specific gene that is preferentially expressed during early stages of male gametogenesis [18], [19]. WRKY34 also contains several consensus MAPK phosphorylation sites at similar positions as WRKY33 (Figure 1A), indicating that WRKY34 might be a MPK3/MPK6 substrate as well.

To determine if WRKY34 can be phosphorylated by MAPKs *in vitro*, we prepared a His-tagged recombinant WRKY34 protein for *in vitro* MAPK phosphorylation assays. WRKY34 can be strongly phosphorylated by activated MPK3 and MPK6 (Figure 1B, upper panel). Without activation by the constitutively active MKK4^DD^/MKK5^DD^, MPK3 weakly phosphorylated WRKY34, whereas MPK6 showed no activity, demonstrating that the activation of MPK3 and MPK6 was important for a high-level phosphorylation of WRKY34. Control reactions with myelin basic protein (MBP) as an artificial substrate confirmed MPK3/MPK6 activation (Figure 1B, lower panel). There are six putative MAPK phosphorylation sites (Ser-87, Ser-91, Ser-98, Ser-108, Ser-274, and Ser-544) within the WRKY34 protein (Figure 1C). We performed site-directed mutagenesis to change these sites from Ser to Ala (WRKY34^SA^). As shown in Figure 1D, the phosphorylation of WRKY34^SA^ protein by MPK3 and MPK6 was greatly reduced, demonstrating that these SP-motifs are the major MPK3/MPK6-phosphorylation sites in WRKY34. The residual phosphorylation of WRKY34^SA^ also indicates the existence of other unidentified minor MAPK phosphorylation site(s) in WRKY34.

### Detection of epitope-tagged WRKY34 protein from *LAT52*-driven transgene at different stages of male gametogenesis

**Figure 2:**
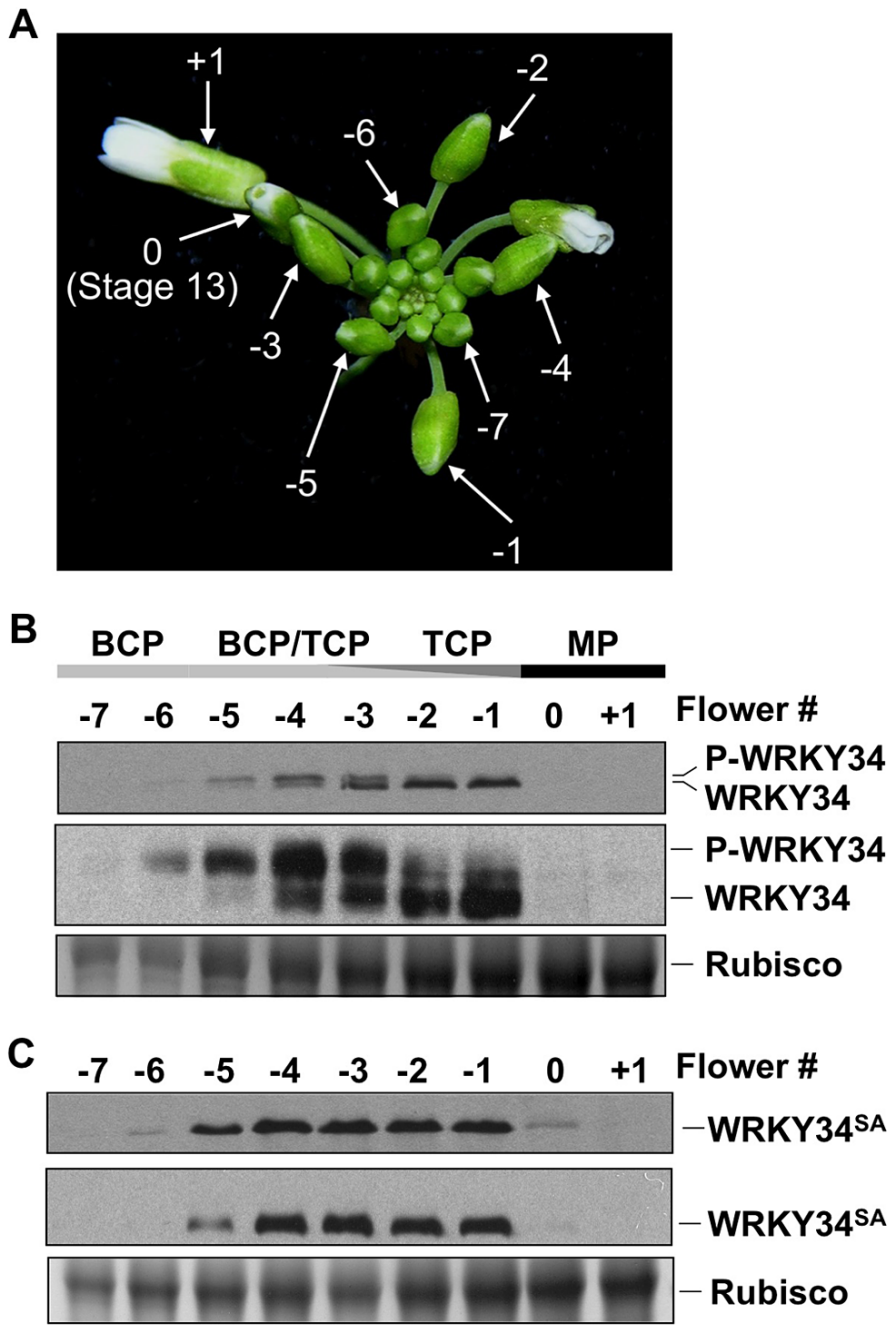
Phosphorylation of WRKY34 *in vivo* during male gametogenesis. (A) Staging of flowers/buds used for WRKY34 protein analysis. Flower at Stage 13, in which anthesis is about to occur, was designated 0. An open flower right after anthesis was designated +1. Younger flowers/buds were designated using negative numbers. (B) Immunoblot and Phos-tag assays of *LAT52* promoter-driven pollen-specific 4myc-WRKY34 protein at different stages of pollen development. The −7 to +1 flowers/buds have pollen at different developmental stages. Black bar indicates flowers or buds containing mature pollen. Dark gray bar indicates buds containing tricellular pollen (TCP). Light gray bar indicates buds containing bicellular pollen (BCP). Levels of 4myc-WRKY34 protein in immunoblot (top panel) and Phos-tag assay (middle panel) were determined using an anti-myc antibody. Protein loading control was confirmed by Coomassie blue staining (bottom panel). (C) Immunoblot (top panel) and Phos-tag assay (middle panel) of pollen-specific 4myc-WRKY34^SA^ protein at different developmental stages. Protein loading control was confirmed by Coomassie blue staining (bottom panel). Each sample was extracted from the same number of flowers/buds at the corresponding stage, which allows the comparison of WRKY34 protein levels in an equal number of developing/mature pollen grains.

To determine whether WRKY34 is phosphorylated by MPK3/MPK6 *in vivo*, we developed an immunoblot protocol to detect WRKY34 protein during male gametogenesis. A four-copy myc tag (4myc) was fused to the N terminus of WRKY34 protein, and a pollen-specific *LAT52* promoter [20] was used to drive the transgene so that the 4myc-tagged WRKY34 protein could be expressed specifically and highly in pollen. Flowers or buds at various stages were collected for immunoblot detection of 4myc-WRKY34 protein in pollen. In this assay, the open flower right after anthesis was designated +1 (Figure 2A). The flower at Stage 13, in which anthesis was about to occur [21], was designated as 0. Buds/flowers at earlier stages were named with negative numbers −1, −2, and so on, according to their relative positions to the number 0 flower (Figure 2A). Under our experimental conditions, as few as 10 flowers/buds were sufficient for protein extraction and the detection of 4myc-WRKY34 protein by immunoblot analysis. The stage of pollen development was determined by DAPI staining of pollen grains from dissected flowers/buds of multiple plants. The +1 and 0 flowers contained mature pollen (MP) grains. The −1 and −2 buds contained homogenous tricellular pollen (TCP). The −3 to −5 buds contained a mixture of TCP and bicellular pollen (BCP), indicating non-uniform development of pollen in these bud stages. The −6 and −7 buds contained solely BCP.

We found that tagged 4myc-WRKY34 protein was first detectable in −6 buds, which contain BCP (Figure 2B, top panel). The absence of 4myc-WRKY34 protein in earlier stages is likely a result of low *LAT52* promoter activity [22]. The 4myc-WRKY34 signal was stronger in more developed buds and reached its peak in −2 and −1 buds with TCP (Figure 2B, top panel). Interestingly, although driven by *LAT52*, a promoter with the strongest activity in mature pollen [22], the 4myc-WRKY34 protein signal was hardly detectable in 0 buds and open flowers (Figure 2B, top panel). The transcripts from *4myc-WRKY34* transgene showed a similar expression pattern, as indicated by RT-PCR (Figure S1). We also tried an immunoblot assay using flowers from *WRKY34 promoter-*driven *4myc-WRKY34* transgenic plants (*P_WRKY34_:4myc-WRKY34*). However, 4myc-WRKY34 protein was not detectable in such samples, which is likely due to low *WRKY34* promoter activity (data not shown). Interestingly, as described later, the *P_WRKY34_:WRKY34-YFP* fusion showed a similar expression pattern as *P_LAT52_:4myc-WRKY34*. Therefore, we conclude that the use of *LAT52* promoter in this assay could represent, at least partially, the native WRKY34 expression and modification pattern.

### WRKY34 is temporally phosphorylated by MAPKs during male gametogenesis

In the immunoblot assay, we noticed that 4myc-WRKY34 showed differential migrations in the SDS-polyacrylamide gel depending on the developmental stage of the flower buds. In −6 buds with BCP, 4myc-WRKY34 protein exhibited a slightly slower migration (Figure 2B, top panel). In −5 to −3 buds with a mixture of BCP and TCP, 4myc-WRKY34 existed as doublets, and the faster moving band gradually accumulated (Figure 2B, top panel). In −1 and −2 buds with TCP, 4myc-WRKY34 protein predominately existed as the faster migrating band (Figure 2B, top panel). These results indicated that WRKY34 protein was modified in BCP, possibly by protein phosphorylation, and the modification is dependent on the pollen's developmental stage. To determine whether the slower migrating band of 4myc-WRKY34 is due to phosphorylation, we performed a Phos-tag mobility shift assay. In this assay, the Phos-tag reagent binds specifically to phosphorylated proteins and slows down their migration in the SDS-polyacrylamide gel [9], [23]. As shown in Figure 2B (middle panel), 4myc-WRKY34 protein was indeed phosphorylated in the BCP of −6 buds and was gradually dephosphorylated in late stages of the male gametogenesis. The phosphorylation of 4myc-WRKY34 was greatly reduced upon pollen maturation at −1, which is followed by complete disappearance of WRKY34 protein in 0 flowers (Figure 2B, top panel).

We then performed immunoblot with *4myc-WRKY34^SA^* transgenic plants to determine if the shifting of protein bands is dependent on the MAPK phosphorylation sites in WRKY34. Although the protein expression pattern is similar to 4myc-WRKY34, the 4myc-WRKY34^SA^ protein showed no band shift in either the immunoblot or Phos-tag assay (Figure 2C). This result further confirmed that WRKY34 was temporally phosphorylated during early pollen development, and the phosphorylation occurred on the MPK3/MPK6-phosphorylation sites delineated in the *in vitro* phosphorylation assay (Figure 1C and 1D).

**Figure 3:**
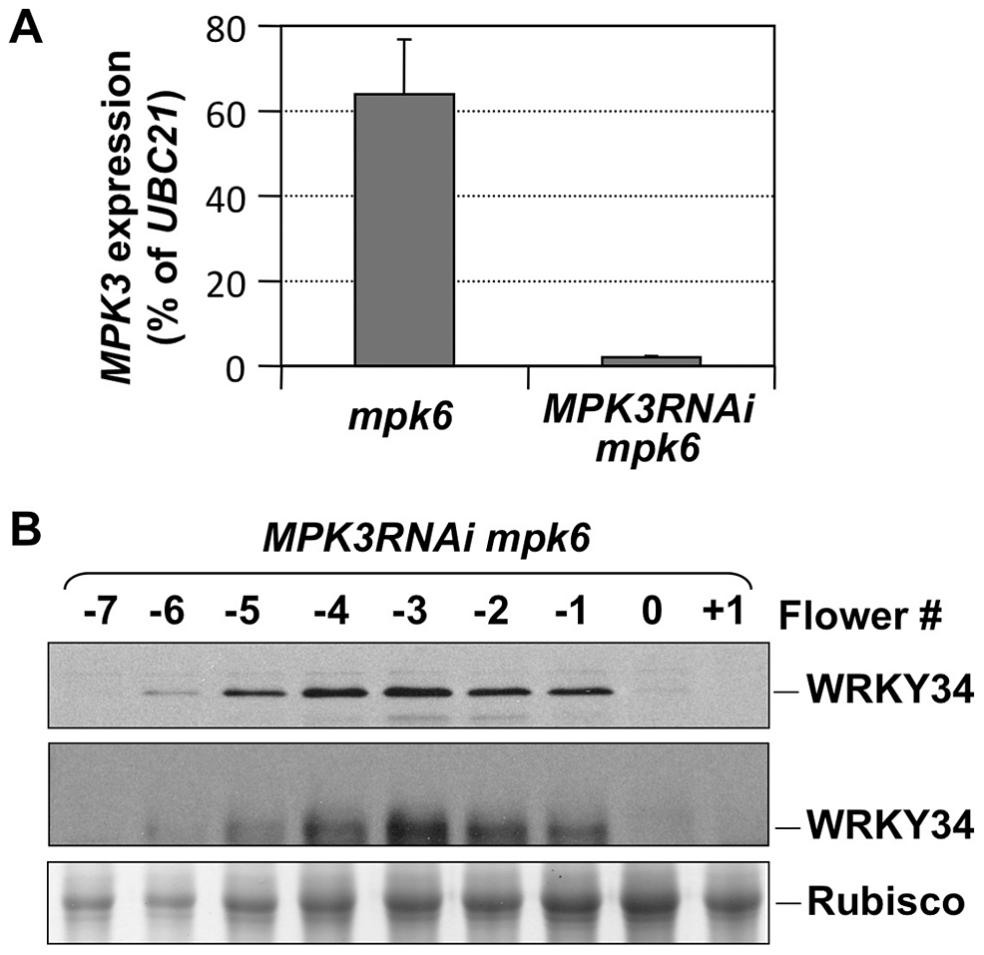
*In vivo* phosphorylation of WRKY34 is dependent on MPK3 and MPK6. (A) *MPK3* expression in *mpk6* and *MPK3RNAi mpk6* pollen grains. Total RNAs were isolated from pollen grains. MPK3 transcript levels were determined using quantitative RT-PCR. Error bars = standard derivation. (B) Immunoblot (top panel) and Phos-tag (middle panel) assays of WRKY34 protein at different stages of *MPK3RNAi mpk6 P_LAT52_:4myc-WRKY34* flower buds. Protein loading control was confirmed by Coomassie blue staining (bottom panel). Each sample was extracted from the same number of flowers/buds at the corresponding stage, which allows the comparison of WRKY34 protein levels in an equal number of developing/mature pollen grains.

To demonstrate that the *in vivo* phosphorylation of WRKY34 during pollen development is carried out by MPK3 and MPK6, we introduced the *4myc-WRKY34* transgene into the *mpk3 mpk6* double mutant background. Since the *mpk3 mpk6* double mutant is embryo lethal [13], we attempted pollen-specific RNAi suppression of *MPK3* in the *mpk6* mutant background. *LAT52* promoter-driven *MPK3RNAi* construct was transformed into the *mpk6* plants. Because of the pollen-specific expression of *MPK3RNAi*, the sporophytic tissues were not affected, which allowed us to obtain the double homozygous *MPK3RNAi mpk6* plants. Real-time qPCR demonstrated that *MPK3* expression in pollen from *MPK3RNAi mpk6* plants was knocked down (Figure 3A). We then performed immunoblot and Phos-tag assays of 4myc-WRKY34 in the *MPK3RNAi mpk6* plants. The mobility shift of 4myc-WRKY34 was abolished in the absence of MPK3 and MPK6 (Figure 3B, top and middle panels). This loss-of-function system demonstrated that the WRKY34 was phosphorylated specifically by MPK3 and/or MPK6. The stability of WRKY34 protein apparently was not affected by the MAPK phosphorylation since mutation of the Ser residues that are phosphorylated by MPK3/MPK6 did not affect the protein expression pattern of WRKY34 during pollen development (Figures 2C).

### *WRKY34* functions redundantly with *WRKY2* in pollen development

Previous studies showed that *WRKY34* is an early pollen gene enriched in UNM and BCP [19] and that mutation of the *WRKY34* gene increases the pollen's tolerance to cold stress [18]. However, the biological function of WRKY34 in pollen development remains unclear. Under our growth conditions, single *wrky34* mutant pollen showed no developmental defect. Since more than 50% of the *WRKY* family members are expressed in the male gametophytes [2], we speculated that there might be functionally redundant *WRKY* member(s) in early pollen development.

**Figure 4:**
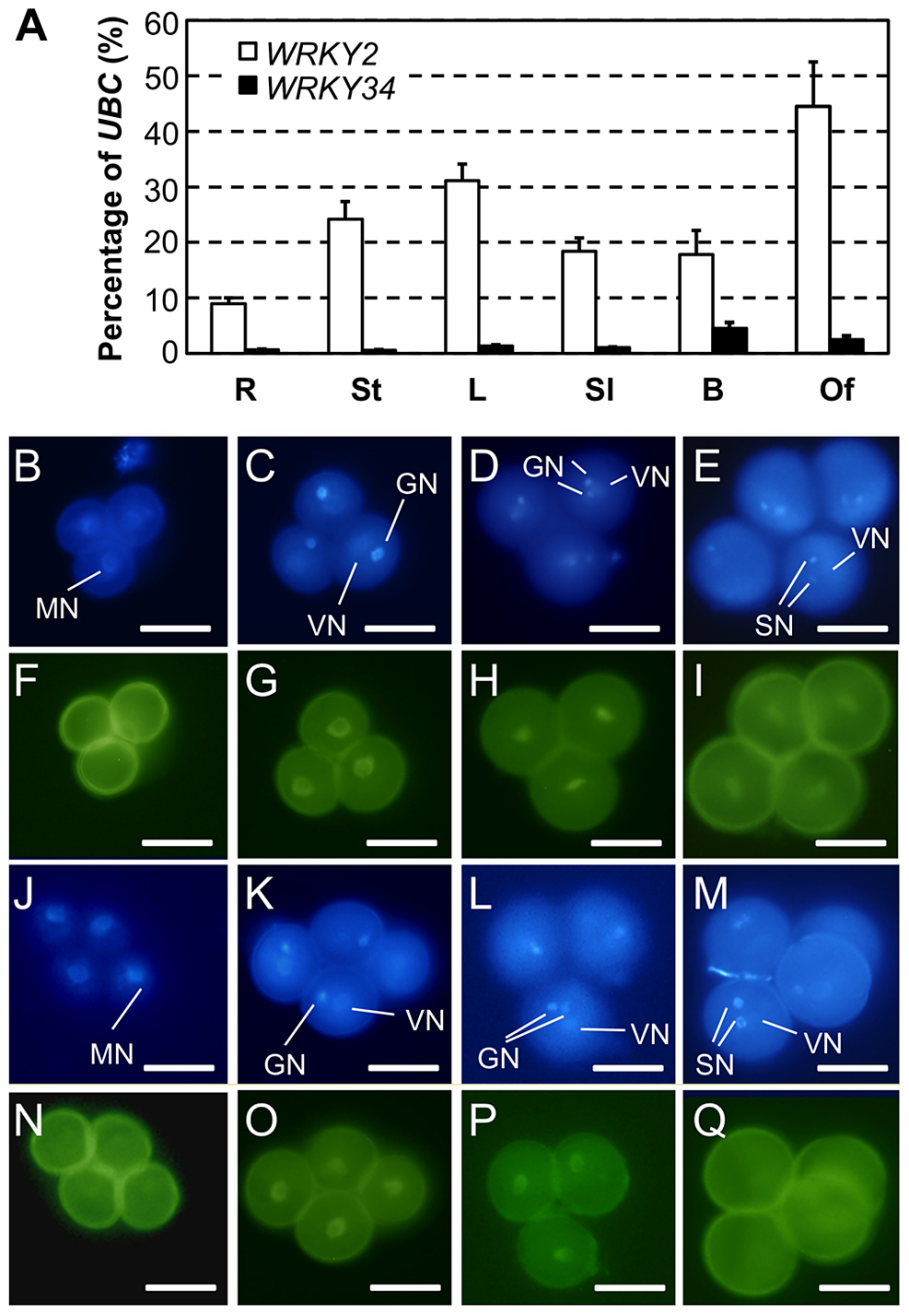
Expression and protein localization of WRKY34 and WRKY2. (A) Quantitative RT-PCR of *WRKY34* and *WRKY2* transcripts in various tissues. R, roots; St, stems; L, leaves; Sl, seedlings; B, buds; and Of, open flowers. Error bars = standard derivation. (B to I) Expression and localization of *WRKY2* promoter-driven WRKY2:YFP fusion protein in pollen. DAPI staining was used to locate nuclei (B to E), and YFP signal reveals the localization of WRKY2:YFP fusion at different developmental stages (F to I**)**. (B and F) UNM stage, no nucleus-localized YFP signal was detected. Vegetative nucleus localized WRKY2:YFP signal was observed at BCP stage (C and G), TCP stage (D and H), and MP stage (E and I). (J to Q) Expression and localization of *WRKY34* promoter-driven WRKY34:YFP fusion protein in pollen. (J to M) DAPI staining signal. (N to Q) YFP signal. (J and N) UNM stage, weak signal was observed in microspore nucleus. Vegetative nucleus localized WRKY34:YFP signal was observed at BCP stage (K and O), and TCP stage (L and P). No YFP signal was observed in MP (M and Q). Note that as the vegetative cell expressed genes, the WRKY2 and WRKY34 fusion YFP signals were not detectable in generative or sperm cells. MN, microspore nucleus. VN, vegetative nucleus. GN, generative nucleus/nuclei. SN, sperm cell nuclei. Bar = 50 µm.

A phylogenetic analysis was used to identify such member(s) (Figure S2). WRKY34 is closely related to WRKY2, a WRKY member expressed in various tissues including male gametophyte [24]. We examined by quantitative RT-PCR the expression patterns of *WRKY34* and *WRKY2* in several tissues. *WRKY34* expression was very low in most examined tissues and was slightly higher in floral buds (Figure 4A). In contrast, WRKY2 showed higher expression in all detected tissues (Figure 4A). To examine the detailed expression patterns of WRKY2 and WRKY34 in pollen at different stages, we fused the *WRKY2* and *WRKY34* genomic sequences, which contain promoter and gene coding region, with YFP. The YFP signal of both fusion proteins was detectable in nuclei, which was consistent with their function as transcription factors. It is also noteworthy that WRKY2- and WRKY34-YFP signals were detectable in the vegetative cell but not in the generative or sperm cells. The *P_WRKY2_:WRKY2-YFP* signal was absent in UNMs (Figure 4B and 4F), while it became significantly higher in BCP nuclei (Figure 4C and 4G). The YFP signal in nuclei was also found in TCP (Figure 4D and 4H) and MP (Figure 4E and 4I). For *P_WRKY34_:WRKY34-YFP*, the nucleus YFP signal was dim in UNM, although it was still distinguishable from the pollen auto-fluorescence (Figure 4J and 4N). The signal was more detectable in BCP (Figure 4K and 4O) and TCP (Figure 4L and 4P). However, in contrast to WRKY2, the WRKY34-YFP signal was absent in MP (Figure 4M and 4Q). These results showed that *WRKY34* and *WRKY2* expression overlaps at the BCP and TCP stages. In addition, the *P_WRKY34_:WRKY34-YFP* expression pattern was similar to the *P_LAT52_:4myc-WRKY34* expression in the immunoblot assay (Figure 2B, top panel). This further indicated that the WRKY34 protein expression pattern was not solely dependent on promoter activity.

**Figure 5:**
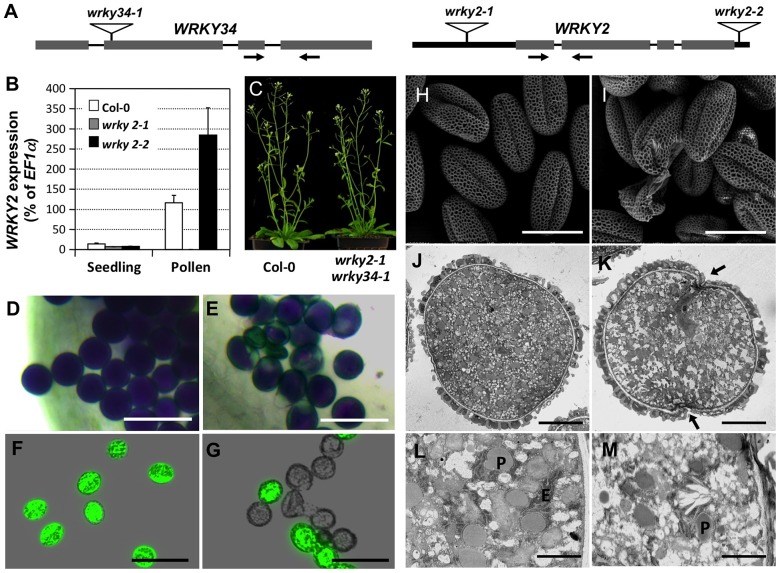
Phenotype of *wrky2-1 wrky34-1* double mutant pollen. (A) Diagram of T-DNA insertion alleles of *wrky2* and *wrky34* mutants. Arrows indicate the positions of RT-qPCR primers. Black bars = untranslated regions (UTRs); gray bars = exons; lines = introns. (B) Quantitative RT-PCR of *WRKY2* expression in wild-type, *wrky2-1*, and *wrky2-2* seedlings and pollen grains. Error bars = standard derivation. (C) Normal vegetative growth and development of *wrky2-1 wrky34-1* double mutant plants. Five-week-old plants are pictured. (D, E) Alexander staining of wild-type (D) and *wrky2-1 wrky34-1* double mutant (E) pollen. Bar = 50 µm. (F, G) Vital staining by FDA of wild type (F) and *wrky2-1 wrky34-1* double mutant (G) pollen. Bar = 50 µm. (H, I) Scanning electron microscopy (SEM) of wild type (H) and *wrky2-1 wrky34-1* double mutant (I) pollen. Bar = 20 µm. (J, K, L, M) Transmission electron microscopy (TEM) of wild type (J, L) and *wrky2-1 wrky34-1* double mutant (K, M) pollen. (J, K) Bar = 5 µm. (L, M) Bar = 1 µm. Arrows in panel K indicate the germination pore with defective intine layer. P, plastid; E, endoplasmic reticulum.

We next obtained a T-DNA insertion line for *WRKY34* (SALK_133019 hereafter *wrky34-1*) and two T-DNA lines for *WRKY2* (Salk_020399 and SAIL_739_F05, hereafter *wrky2-1* and *wrky2-2*, respectively) (Figure 5A). *wrky34-1* was reported to be a null mutant [18]. We performed quantitative RT-PCR to examine *WRKY2* expression in wild-type, *wrky2-1*, and *wrky2-2* pollen. The result showed that the expression of *WRKY2* was moderately knocked down in seedlings of both alleles (Figure 5B). However, in pollen, *WRKY2* expression was almost completely knocked out in *wrky2-1* but not in *wrky2-2* (Figure 5B). Therefore, we crossed *wrky34-1* with *wrky2-1* to generate the *wrky2-1 wrky34-1* double mutant and then examined the *wrky2-1 wrky34-1* double mutant pollen function by reciprocal crosses using combinations of heterozygous mutants and wild type (Table 1).

**Table 1:** Transmission of *wrky2-1* and *wrky34-1* single and double mutant alleles.

♂	♀	Transmission segregation	Ratio	*p-value*
*wrky34-1^+/−^*	*WT*	*WRKY34: wrky34-1*	207∶196	0.6188
*wrky2-1^+/−^*	*WT*	*WRKY2: wrky2-1*	156∶121	0.0355
*wrky2-1^+/−^ wrky34-1^−/−^*	*WT*	*WRKY2 wrky34-1:wrky2-1 wrky34-1*	**251∶11**	<0.0001
*wrky2-1^−/−^ wrky34-1^+/−^*	*WT*	*wrky2-1 WRKY34:wrky2-1 wrky34-1*	**154∶2**	<0.0001
*WT*	*wrky2-1^−/−^ wrky34-1^+/−^*	*wrky2-1 WRKY34:wrky2-1 wrky34-1*	77∶93	0.219

Crosses were performed using plants of indicated genotype. The genotypes of F1 progenies were determined by PCR genotyping, which was used to determine the transmission of pollen of different genotypes. Bold numbers indicate significant aberrant transmission ratios from the expected ratio of 1∶1.

The male transmission of the mutant alleles was normal when pollen grains from either *wrky2-1^+/−^* or *wrky34-1* ^+/−^ plants were used as pollen donors, suggesting that single mutations of either *WRKY34* or *WRKY2* had no effect on the function of pollen (Table 1). However, when using *wrky2-1^+/−^ wrky34-1^−/−^* or *wrky2-1^−/−^ wrky34-1^+/−^* plants as the male parents, we observed that the transmission of *wrky2-1 wrky34-1* pollen was significantly reduced (0.04∶1 for pollen from *wrky2-1^+/−^ wrky34-1^−/−^* plants and 0.01∶1 for pollen from *wrky2-1^−/−^ wrky34-1^+/−^* plants, instead of the expected 1∶1, *p*-value<0.0001) (Table 1). This result suggested that *WRKY34* and *WRKY2* are important for pollen function but also that a portion of the double mutant pollen grains remained functional. The transmission of *wrky2-1 wrky34-1* female gametophytes was normal (Table 1), indicating that the female gametophyte function was not affected.

### Phenotype of *wrky2-1 wrky34-1* double mutant pollen

Because of the leaky transmission of *wrky2-1 wrky34-1* pollen, we were able to obtain *wrky2-1 wrky34-1* homozygous double mutant plants at low frequency. Morphologically, the double mutant plant was indistinguishable from the wild type (Figure 5C). To examine the development of *wrky2-1 wrky34-1* pollen, we used Alexander's staining to distinguish normal and aborted pollen [25]. In this assay, the cytoplasm of normal pollen should show a purple color and the pollen wall a distinctive green color. Pollen grains from wild-type plants were viewed as full, round, purple-stained grains (Figure 5D). In contrast, a portion of *wrky2-1 wrky34-1* pollen exhibited aberrant morphology and green color (28% abortion, n = 200), which indicated impaired pollen development of the double mutant (Figure 5E). We then performed fluorescein diacetate (FDA) staining to check the viability of *wrky2-1 wrky34-1* pollen (Figure 5F and 5G). In comparison with wild-type pollen grains (96% viable), the majority of *wrky2-1 wrky34-1* pollen failed to show FDA fluorescence and therefore was likely to be dead (67%).

The non-viable rate in FDA staining was higher than that in the Alexander staining, indicating that FDA is a more sensitive viability assay. There were *wrky2-1 wrky34-1* pollen grains with a small patch that failed to be stained using Alexander staining (Figure 5E). They were classified as viable pollen, but might be non-viable. In contrast, FDA staining, which is dependent on both cellular esterase activity and plasma membrane integrity, gave much clearer results. For this reason, FDA staining was used for all the other experiments. We next stained the developing pollen at earlier stages with FDA. The lethality of *wrky2-1 wrky34-1* pollen was first identifiable in -6 buds with BCP, and the percentage of lethal pollen increased following pollen development (Figure S3). The onset of pollen death in *wrky2-1 wrky34-1* double mutant correlates with the appearance of WRKY34 protein in BCP and TCP stages (Figure 2B, top panel), suggesting the requirement of these two WRKYs at these developmental stages. There are two possible reasons for the lower percentages of FDA positive pollen at early developmental stages and then the gradual increase in FDA positive pollen in the wild type (Figure S3). Firstly, the tapetal cell layer surrounding the developing pollen could reduce the efficiency of FDA staining at the early stage. Secondly, dissection and squeezing to release pollen from the anther and tapetum might damage the immature pollen. Side-by-side comparison revealed that the FDA positive pollen from *wrky2-1 wrky34-1* plants continued to drop (Figure S3), indicating the loss of viability of *wrky2-1 wrky34-1* mutant pollen.

We also examined the ultrastructure of *wrky2-1 wrky34-1* pollen using scanning electron microscopy (SEM). Both wild-type and *wrky2-1 wrky34-1* pollen appeared to have normal pollen wall structures (Figure 5H and 5I). In contrast to the uniformly shaped wild-type pollen grains (Figure 5H), the *wrky2-1 wrky34-1* pollen grains were a mixture of shapes, including normal shaped pollen, collapsed pollen, and ruptured pollen remnant (Figure 5I). This indicated that the development of *wrky2-1 wrky34-1* pollen was defective. Further analysis with transmission electron microscopy (TEM) confirmed the abnormal ultrastructure of *wrky2-1 wrky34-1* pollen. Consistent with the cytological staining results, a portion of the *wrky2-1 wrky34-1* pollen was collapsed with leaky cytoplasm content (Figure S4). Furthermore, for the majority of *wrky2-1 wrky34-1* pollen that exhibited similar exterior appearance as wild-type pollen, the intracellular ultrastructure was different from that of the wild-type pollen (Figure 5J to 5M). The numbers of plastids and endoplasmic reticulum (ER) were reduced in *wrky2-1 wrky34-1* pollen grain. In addition, the intine layer was discontinuous and undulated at the germination pore of the double mutant pollen grain (Figure 5K and 5M).

### Pollen tube growth is impaired in *wrky2-1 wrky34-1*

**Figure 6:**
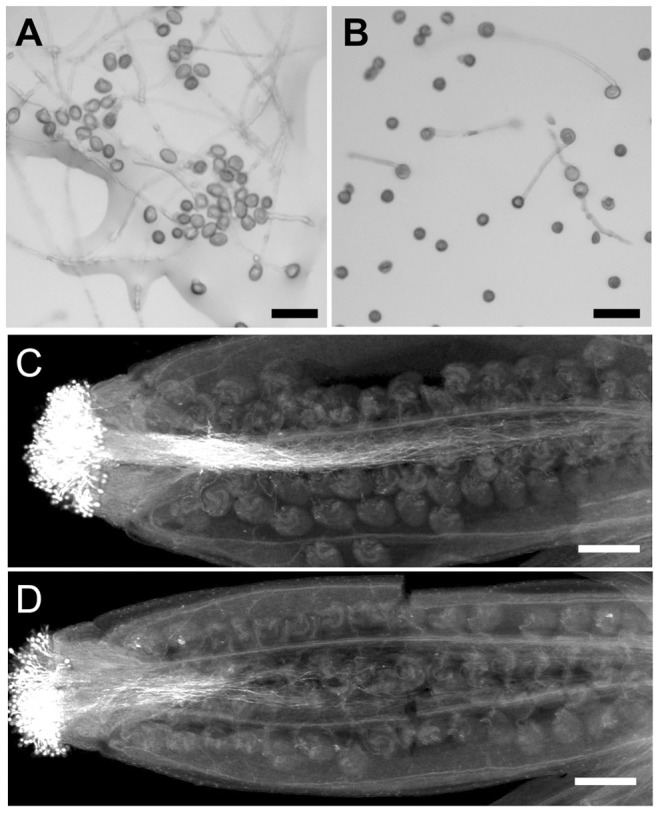
Pollen germination and pollen tube growth are defective in *wrky2-1 wrky34-1*. *In vitro* pollen germination of wild type (A) and *wrky2-1 wrky34-1* double mutant (B) pollen. Bars = 50 µm. Aniline blue staining of wild type pistils 8 hours after pollination with wild type (C) and *wrky2-1 wrky34-1* double mutant (D) pollen. Bar = 200 µm.

**Figure 7:**
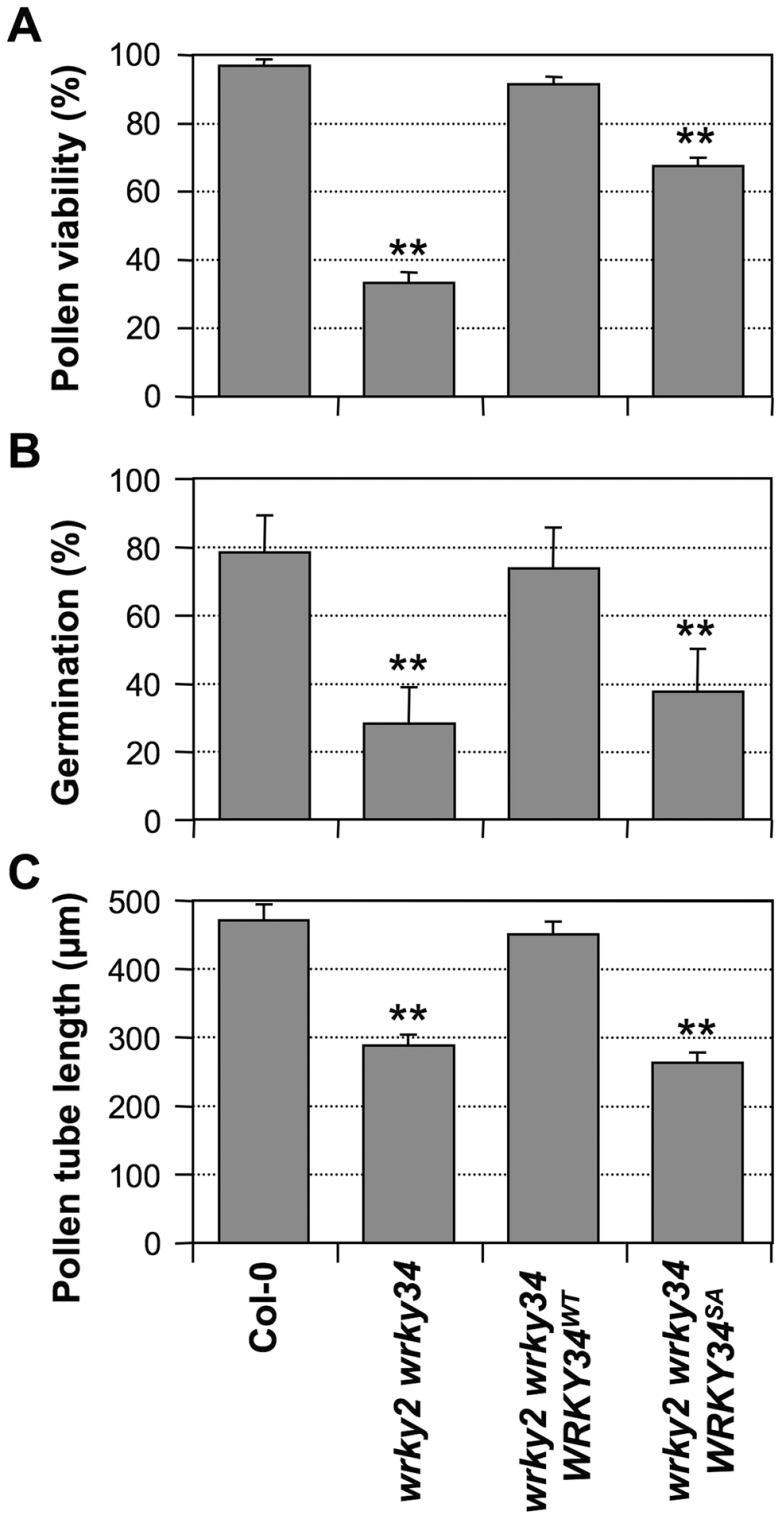
Complementation of *wrky2-1 wrky34-1* double mutant pollen phenotypes by *WRKY34^WT^* and *WRKY34^SA^*. Pollen viability ratio by FDA staining **(**A), *in vitro* pollen germination ratio (B), and average pollen tube length (C) in Col-0, *wrky2-1 wrky34-1*, *wrky2-1 wrky34-1 P_WRKY34_:WRKY34^WT^*, and *wrky2-1 wrky34-1 P_WRKY34_:WRKY34^SA^* plants. Error bar = standard error. Double asterisks indicate statistically very significant difference from wild-type pollen (*p-value*<0.01).

In addition to a pollen developmental defect, the *in vitro* germination assay revealed that the *wrky2-1 wrky34-1* double mutant was defective in pollen function. In our assays, the average germination ratio of wild-type pollen was 78% (Figures 6A and 7B), while only 28% of *wrky2-1 wrky34-1* pollen was capable of germination under the same conditions (Figures 6B and 7B). The reduction in pollen germination appears to be a result of reduced pollen viability. For the *wrky2-1 wrky34-1* pollen that germinated, the pollen tube length was significantly shorter than the length of wild-type pollen tubes (Figure 6A, 6B, and 7C). The average pollen tube length was 471 µm in the wild type and 288 µm in *wrky2-1 wrky34-1* double mutant at 7 hours after germination *in vitro*, representing a 40% reduction in length in the double mutant pollen tubes. Pollination analysis followed by aniline blue staining further demonstrated that the *wrky2-1 wrky34-1* double mutant was defective in pollen germination and pollen tube growth *in vivo* (Figure 6C and 6D). Since WRKY34 protein was degraded before pollen maturation (Figure 2B, top panel), we speculated that the reduced germination and tube growth of *wrky2-1 wrky34-1* pollen were not an indication of a requirement of WRKY2/WRKY34 in these two processes but rather a result of weak pollen due to impaired development, which was also evident based on the TEM observation (Figure 5J to 5M).

### Phosphorylation by MAPKs is required for WRKY34 function *in vivo*

To test whether phosphorylation of WRKY34 is important for its function in pollen development, we performed genetic complementation of *wrky2-1 wrky34-1* pollen using *WRKY34 promoter*-driven *4myc-WRKY34^WT^* or *4myc-WRKY34^SA^*. Pollen from T2 homozygous progenies with a transgene expression level similar to wild type was selected and examined (Figure S5). *P_WRKY34_:4myc-WRKY34^WT^ wrky2-1 wrky34-1* pollen showed viability, germination, and pollen tube growth similar to wild-type pollen (Figure 7), indicating that the *P_WRKY34_: 4myc-WRKY34^WT^* transgene can complement the *wrky2-1 wrky34-1* pollen phenotype. In contrast, the function of *P_WRKY34_:4myc-WRKY34^SA^* transgene was significantly compromised. The viability ratio of *P_WRKY34_:4myc-WRKY34^SA^ wrky2-1 wrky34-1* pollen was partially rescued to 67% from 33% of the *wrky2-1 wrky34-1* pollen, which was significantly lower than wild-type (97%) and *P_WRKY34_:WRKY34^WT^* complemented pollen (90%) (*p-value* <0.01) (Figure 7A). The double mutant pollen germination rate (28%) was only slightly rescued by *P_WRKY34_:4myc-WRKY34^SA^* (37%), while it was fully complemented by *P_WRKY34_:4myc-WRKY34^WT^* (73%), which was similar to wild-type pollen (78%) (*p-value* <0.01) (Figure 7B). Furthermore, the average pollen tube length of *P_WRKY34_:4myc-WRKY34^SA^ wrky2-1 wrky34-1* pollen (263 µm) was about the same as the double mutant (288 µm) and much shorter than the wild-type (471 µm) and *P_WRKY34_:4myc-WRKY34^WT^ wrky2-1 wrky34-1* pollen (451 µm) (*p-value* <0.01) (Figure 7C). These results indicated that although the pollen lethality of the double mutant was partially rescued by WRKY34^SA^, the pollen function was still abnormal. As a result, we conclude that the phosphorylation of WRKY34 by MPK3/MPK6 is important for the function of WRKY34 protein *in vivo*.

Although the native promoter-driven *WRKY34* transgene (*P_WRKY34_:4myc-WRKY34^WT^*) could fully complement the *wrky2-1 wrky34-1* mutant, immunoblot analysis failed to detect the tagged WRKY34^WT^ protein in the inflorescences or floral buds in these rescued lines (data not shown), which is most likely a result of the low expression level of the native promoter (Figure 4). To exclude the possibility that mutation of multiple Ser to Ala in WRKY34 altered its general functionality such that the WRKY34^SA^ cannot bind or has reduced DNA-binding activity, we compared the W-box binding activity of the recombinant WRKY34 and WRKY34^SA^ using electrophoretic mobility shift assay (EMSA). As shown in Figure S6, there was no difference in the W-box binding activity and specificity of WRKY34 after the Ser-to-Ala mutation, suggesting that the reduced functionality of WRKY34^SA^ is not a result of a general loss of WRKY34 function.

### *MPK6* belongs to the same genetic pathway of *WRKY34* and *WRKY2*

Based on these results, the phosphorylation by MPK3/MPK6 is important for the function of WRKY34 in pollen development and function. Due to the embryo lethality of *mpk3 mpk6* double zygotes [13], we cannot analyze the phenotype of pollen grains from the double homozygous plants. The *mpk3 mpk6* double mutant pollen from *mpk3^+/−^ mpk6^−/−^* or *mpk3^−/−^ mpk6^+/−^* plants, although it exhibited altered transmission, did not show any developmental defects like *wrky2-1 wrky34* pollen [26]. We speculate that in *mpk3 mpk6* pollen the unphosphorylated WRKY34 and WRKY2 each retained basal level function, which kept the *mpk3 mpk6* pollen above the threshold of visible developmental defects. This is consistent with the finding that WRKY34^SA^ mutant protein can partially complement the *wrky2-1 wrky34-1* mutant pollen. Alternatively, MPK3 or MPK6 protein carried over from the microspore mother cells of *mpk3^+/−^ mpk6^−/−^* or *mpk3^−/−^ mpk6^+/−^* plants, which have at least one good copy of *MPK3* or *MPK6*, could be sufficient to support the development of *mpk3 mpk6* pollen. It is known that MAPKs are very stable proteins in cells.

**Figure 8:**
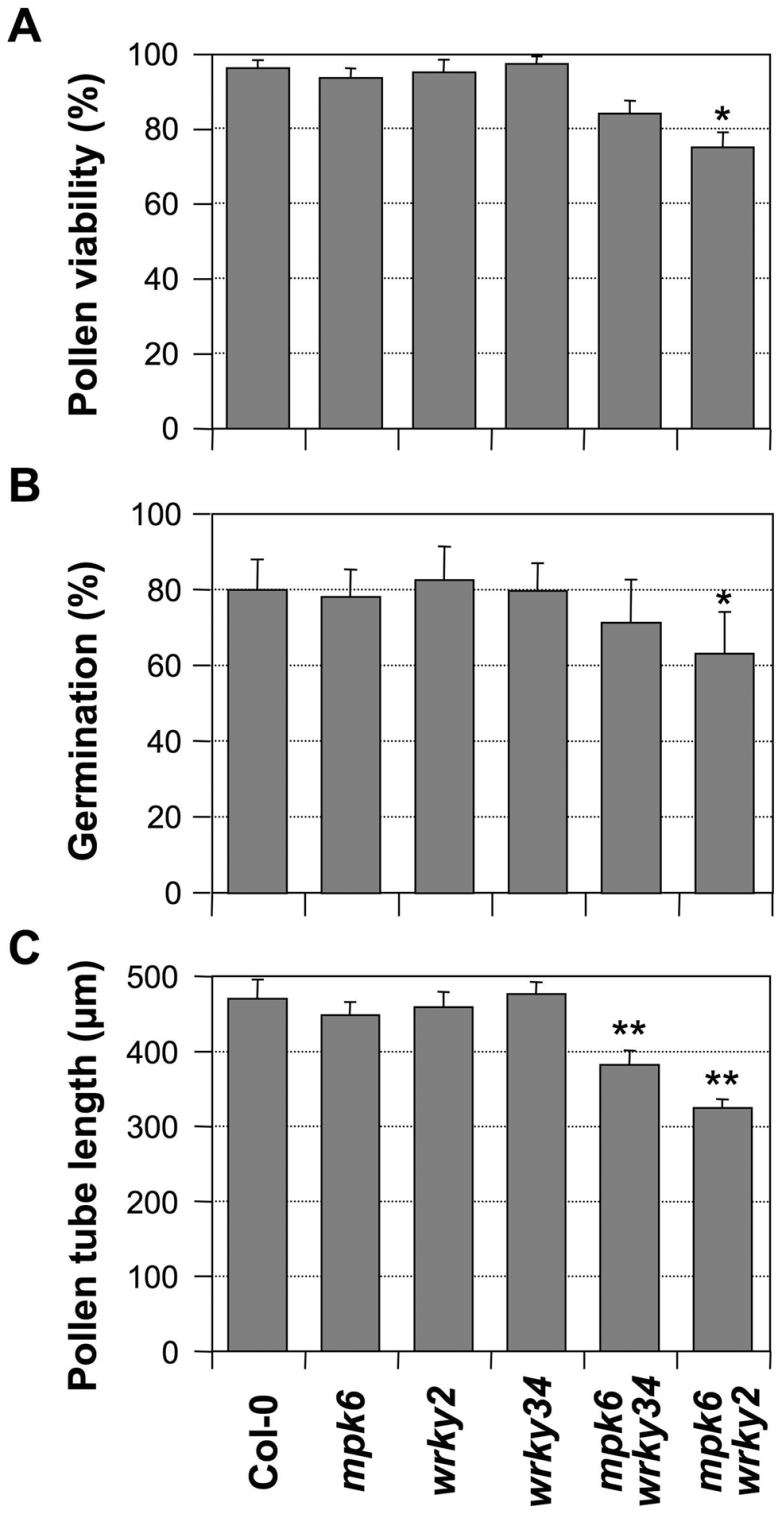
Phenotype of *mpk6 wrky34-1* and *mpk6 wrky2-1* pollen. Pollen viability ratio by FDA staining (A), *in vitro* pollen germination ratio (B), and average pollen tube length (C) in Col-0, *mpk6*, *wrky2-1*, *wrky34-1*, *mpk6 mpk34-1*, and *mpk6 wrky2-1* plants. Error bar = standard error. Single asterisks indicate statistically significant difference from wild-type pollen (0.01<*p-value*<0.05). Double asterisks indicate very significant difference from wild-type pollen (*p-value*<0.01).

Although both MPK3 and MPK6 are involved in pollen function, MPK6 apparently is more important, as indicated by its much higher expression in pollen (www.genevestigator.com). Therefore, we speculate that the double mutation of *mpk6* and *wrky34-1* (or *wrky2-1*), in which the pollen produced a single WRKY protein with reduced phosphorylation, might result in a weak phenotype in pollen development. As shown in Figure 8, both *mpk6 wrky34-1* and *mpk6 wrky2-1* pollen showed developmental and functional defects that were similar to the *wrky34-1 wrky2-1* double mutant pollen. The pollen viability was 84% in *mpk6 wrky34-1* and 75% in *mpk6 wrky2-1*, respectively, which indicated moderate pollen lethality in the double mutants (*p-value*<0.05) (Figure 8A). In accordance, the pollen germination rate was also decreased slightly from an average of 80% of wild-type pollen to 71% of *mpk6 wrky34-1* and 63% of *mpk6 wrky2-1* (*p-value*<0.05) (Figure 8B). Furthermore, the average pollen tube lengths of *mpk6 wrky34-1* and *mpk6 wrky2-1* was significantly reduced to 382 µm and 324 µm, respectively, in comparison with the 470 µm of wild-type pollen tubes (*p-value*<0.01) (Figure 8C). This result indicated that the *mpk6 wrky34-1* and *mpk6 wrky2-1* pollen function was affected and confirmed that *MPK6* belongs to the same genetic pathway as *WRKY34* and *WRKY2*.

## Discussion

In this report, we demonstrate that WRKY34, a pollen-specific WRKY transcription factor, is a substrate of MPK3/MPK6. WRKY34 is temporally phosphorylated during early male gametogenesis and is dephosphorylated right before pollen maturation. The phosphorylation of WRKY34 by MPK3/MPK6 is important for its function *in vivo*. WRKY34, together with WRKY2, is required for male gametogenesis. Mutation of both *WRKY34* and *WRKY2* greatly reduces the viability of pollen, which is associated with reduced germination and pollen tube growth, both *in vitro* and *in vivo*. Taken together, we conclude that WRKY34/WRKY2 transcription factors play an important role downstream of the MPK3/MPK6 cascade in pollen development and function.

### Differential expression of substrates allows the MPK3/MPK6 cascade to control different biological processes

A long-standing question is how a MAPK cascade confers signaling specificity in diverse biological events. In yeast and mammals, the mechanisms to maintain signaling specificity of MAPKs include 1) cell-type specificity of other signaling components in the pathway, such as receptors, scaffolding proteins, and MAPK substrates [27]–[29]; 2) kinetics in signaling strength resulting in distinct outcomes [30]; and 3) cross-pathway suppression of downstream components [31]–[33]. However, in plants, such mechanisms have not been well studied. In *Arabidopsis*, MPK3 and MPK6, two of the best-characterized MAPKs, function together in diverse biological processes, including plant growth, development, and response to environmental stimuli [12]–[14], [16], [26], [34], [35]. Differentially expressed substrates could help maintain the functional specificity of the activated MPK3/MPK6 signaling cascade in different cells/tissues. In response to pathogen attacks, MPK3 and MPK6 are activated and phosphorylate a subset of ACC synthase (ACS) isoforms to induce ethylene biosynthesis [12]. The pathogen responsive MPK3/MPK6 cascade also induces phytoalexin biosynthesis through the activation of downstream the WRKY33 substrate [9], [36]. In stomatal development, MPK3/MPK6 phosphorylates SPEECHLESS, a basic helix-loop-helix transcription factor that is specifically expressed in stomatal lineage cells and negatively regulates stomatal formation [10], [13]. In different biological processes, MPK3 and MPK6 are able to phosphorylate different WRKY homologs, e.g. WRKY33 and WRKY34 in plant defense and pollen development, respectively. Differential tissue/cell-specific expression of WRKY33 and WRKY34 allows the MPK3/MPK6 cascade to control different biological processes.

### WRKY transcription factors share common signaling components in different biological processes

WRKY transcription factors are one of the largest families of transcriptional regulators in plants [17]. Transcriptional regulation by WRKY members is an integral part of signaling networks that modulate many biological processes, most notably in response to diverse biotic and abiotic stresses [37]. WRKY transcription factors also have been implicated in plant growth and development processes, including senescence, seed development, and embryogenesis. For instance, WRKY53 binds to the promoters of a set of senescence-associated genes, and the overexpression or knockdown of *WRKY53* gene lead to an altered senescence phenotype [38]. In seed, a WRKY transcription factor, MINISEED3 (MINI3), recruits a nuclear localized protein SHB1 to activate gene expression, which regulates endosperm proliferation and seed cavity enlargement [39]. The WRKY23 transcription factor is needed for proper root growth and development by stimulating the local biosynthesis of flavonols, which is dependent on auxin through the AUXIN RESPONSE FACTOR 7 (ARF7) and ARF19 transcriptional response pathway [40]. Despite these recent discoveries, it is still unclear whether WRKY transcription factors share similar regulatory networks between environmental responses and developmental processes. Our results suggest that the MPK3/MPK6 signaling module could act as a molecular hub to integrate different signaling networks of WRKY transcription factors, although the upstream signaling cues are different.

MPK3/MPK6 and WRKY34 also may integrate stress and developmental signaling in pollen. WRKY34 is involved in cold sensitivity in mature pollen, where it regulates expression of cold-specific transcription factors (CBF) [18]. MPK6 is rapidly activated by cold stress. Furthermore, MPK6 signaling is functionally involved in cold and salt stress responses [41]. It is therefore possible that MPK3 and MPK6 may be involved in the WRKY34-mediated cold tolerance in pollen. However, the MPK6 activity is positively related with cold tolerance, while WRKY34 seems to be a negative regulator in this process. More details are required to interpret the role of MPK3/MPK6-WRKY34 signaling module in pollen cold tolerance.

*WRKY2* plays a redundant role with *WRKY34* in pollen development. Unlike *WRKY34*, *WKRY2* is expressed in various tissues (Figure 4A) and is likely to play pleiotropic roles in plant development. For example, the involvement of WRKY2 in embryogenesis and ABA-mediated seed germination has been reported [24], [42]. In zygote, WRKY2 directly activates the transcription of WUSCHEL RELATED HOMEOBOX (WOX) genes to regulate polar organelle localization and asymmetric division [24]. Given that the *mpk3 mpk6* double mutant is embryo lethal [13], it is possible that the MAPK signaling cascade is involved also in the regulation of the WRKY2-WOX signaling pathway. Comparative analysis of WRKY2 activation by MPK3/MPK6 in pollen and embryogenesis would provide further insights into the regulation of WRKY transcription factors in diverse biological processes.

### Temporal regulation of WRKY transcription factors at multiple levels

Based on the transcriptomic profiles, two periods of temporal gene expression are defined in pollen development, an early phase and a late phase. Expression of “early genes” occurs after meiosis and declines toward pollen maturation, while “late genes” are preferentially expressed in TCP and MP stages [2], [3]. The vegetative cell early-late transcriptome transition occurs mainly between the BCP and TCP stages, which exhibit not only a significantly reduced number of expressed genes but also a major shift in mRNA populations [2]. WRKY34 has been identified as an “early gene”, and its expression is suppressed by several MIKC* MADS box transcription factors during pollen maturation [5], [19]. In this report, we found that WRKY34 from *LAT52*-driven transgene is phosphorylated at the BCP stage and becomes dephosphorylated at the TCP stage. The phosphorylation of WRKY34 is important for its biological function in male gametogenesis. Therefore, we propose that, besides the regulation at the transcriptional level, the post-translational modifications by MAPKs also plays a critical role in controlling the activity of this WRKY transcription factor, especially during early and late phase transition.

The abundance of WRKY34 in early pollen development is regulated at both post-transcriptional and post-translational levels. In our assays, even though driven by *LAT52*, a promoter specific at later pollen stages [20], *WRKY34* transcript was barely detectable in mature pollen (Figure S1), suggesting potential regulation of *WRKY34* transcripts at the mRNA stability level. Moreover, despite the presence of *WRKY34* transcripts at the TCP stage (Figure S1), WRKY34 protein was absent in MP (Figure 2B, top panel), indicating rapid protein degradation in the process. In support of this conclusion, the abundance of the WRKY34-YFP protein from transgene driven by native *WRKY34* promoter showed a similar pattern, as indicated by the YFP fluorescence (Figure 4). This further demonstrates that the abundance of WRKY34 protein is regulated at multiple levels and is not solely dependent on promoter activity. The protein stability of WRKY34 apparently is not associated with its phosphorylation state, since the abundance of WRKY34^SA^, an unphosphorylatable form of WRKY34, followed the same pattern as WRKY34^WT^ protein (Figure 2C). Therefore, there should be a protein degradation pathway regulating WRKY34 protein abundance at late pollen stages that is independent of MPK3/MPK6. WRKY2 protein appeared to be more stable in mature pollen (Figure 4). Pollen development involves dynamic transition of gene expression profiles, which requires rapid control of the transcriptional factors involved. The regulation of WRKY34 activity at multiple levels may reflect the complexity of the regulation of key transcription factors in this process.

## Materials and Methods

### Plant materials and growth conditions

*Arabidopsis thaliana* Columbia ecotype (Col-0) was used as the wild type. T-DNA insertion alleles of *WRKY34* (SALK_133019) and *WRKY2* (Salk_020399 and SAIL_739_F05) were obtained from the Arabidopsis Biological Resource Center (ABRC). Seeds were surface sterilized and imbibed at 4°C for 3 days, then plated on half-strength Murashige and Skoog medium with 0.45% Phytagar. Plates were incubated in a tissue culture chamber at 22°C under continuous light (70 µE m^−2^ s^−1^) for 7 days. Seedlings were then transplanted to soil and grown in the greenhouse with a 16-h-light/8-h-dark cycle.

In PCR-based genotyping, the presence of the T-DNA and wild-type alleles was detected using LBa1 (5′-TGGTTCACGTAGTGGGCCATCG-3′) and two gene-specific primers: *WRKY2-LP* (5′-TTTTCTTTTTCACACGTTAAGCC-3′) and *WRKY2-RP* (5′-TGTTAGAACACGAATCACCCC-3′) for the *wrky2-1* mutant, *WRKY34-LP* (5′-AGCTTGAGCCCAAGTTAAAGC-3′) and *WRKY34-RP* (5′-GCATGTCTTGGCCAGTACCGGATG-3′) for the *wrky34-1* mutant.

### Molecular cloning and transformation

To generate the binary vector with the *LAT52* promoter overexpression cassette, a modified version of pBI121 [9] was digested with HindIII and XhoI to replace the CaMV *35S* promoter with the *LAT52* promoter. To generate the *P_LAT52_*-driven *4myc-WRKY34* overexpression construct (*P_LAT52_:4myc-WRKY34*), we amplified the *WRKY34* cDNA by using primers *WRKY34-F* (5′-CATATGGCTGGTATTGATAATAAAGCTGCTG-3′) and *WRKY34-B* (5′-ACTAGTCAATATCTGTCGTAATCTACTCAACATCTCTCTG-3′). The PCR fragment was cloned into a modified pBlueScript II KS vector with a four-copy myc epitope tag coding sequence at the 5′-end [9] to generate pBS-*4myc-WRKY34* construct. The *4myc-WRKY34* fragment was then cloned into the pBI-*P_LAT52_* vector using SpeI and XhoI sites.

To generate *WRKY34^SA^* cDNA, mutations were introduced into the pBS-*4myc-WRKY34* construct by Quick Change site-directed mutagenesis [43], [44]. Primers used were as following, with mutated residues in lower case: *WRKY34-S91A* (5′-TCTCTTCTCCTGGTCTTgccCCTGCAACTCTGTTAGAG-3′), *WRKY34-S87/91A* (5′-ATCTCTgcTCCTGGTCTTgccCCTGCAACTCTGTTAGAG-3′), *WRKY34-S98A* (5′-CTCTGTTAGAGgcTCCTGTTTTCCTCTC-3′), *WRKY34-S108A* (5′-CTCAAACCCTTTGCTAgCTCAACAACCGGGAAG-3′), *WRKY34-S544A* (5′-GAAGGTGGAACCAGTGgCACCACAACAGGGAC-3′), and their reverse complementary primers. WRKY34^SA^, with all six Ser residues mutated to Ala residues, was generated by five successive mutagenesis steps and verified by sequencing. To generate the *P_LAT52_:4myc-WRKY34^SA^* construct, the *WRKY34^SA^* fragment was cloned into the pBI-*P_LAT52_* vector using SpeI and XhoI sites.

To generate the pollen-specific *MPK3RNAi* construct, the *MPK3RNAi* sequence, as described previously [13], was cloned into the pBI-*P_LAT52_* vector between the SpeI and XhoI sites. The construct was introduced into the *mpk6-2* mutant [12], and homozygous transgenic plants were identified as *MPK3RNAi mpk6*. To generate the *P_LAT52_: 4myc-WRKY34* overexpression construct with a BASTA selection marker for transformation of a *MPK3RNAi mpk6* plant, the *P_LAT52_:4myc-WRKY34* cassette was amplified and partially digested with ApaI and BamHI and then cloned into the pGreenII vector [45]. To generate *P_WRKY34_:WRKY34-YFP* and *P_WRKY2_:WRKY2-YFP* constructs, genomic fragments of *WRKY34* and *WRKY2* were amplified. PCR products and pGreenII-YFP plasmid were digested by XhoI and EcoRV, and ligation was performed. All the binary vectors described below were transformed into *Agrobacterium* strain GV3101. *Arabidopsis* transformation was performed by the floral dip procedure [46], and transformants were identified by screening for kanamycin or BASTA resistance.

### Cytological and phenotypic analyses

Fluorescence microscopy was performed with an Olympus IX70 inverted microscope with an ORCA digital camera. Pollen viability was examined using Alexander staining [25]. Pictures were taken on an Olympus Vanox AHBT3 upright microscope with a color digital camera. The FDA staining assay was performed as described [47]. DAPI was used to stain vegetative and generative/sperm nuclei to determine the pollen development. For FDA or DAPI staining of developing pollen, floral buds at each stage were carefully dissected under stereoscope. Anthers were isolated and transferred to a drop of FDA or DAPI solution. A fine needle was used to gently break the anthers, a cover slip was then used to carefully squeeze the anthers to release the pollen. For SEM, fresh pollen grains were coated directly with platinum and observed on an FEI Quanta 600 FEG Extended Vacuum Scanning Electron Microscope. Pollen germination assays were performed as described [48]. For pollen tube length measurements at 7 hour after germination, at least 100 pollen tubes in each sample were determined using ImageJ software [49]. The presented data are an average of 3 biological repeats.

### *In vitro* phosphorylation assay

For purification of recombinant WRKY34 and its mutant proteins, the *WRKY34^WT^* and *WRKY34^SA^* cDNAs were cut from the pBS-*4myc-WRKY34* constructs with NdeI/SpeI and ligated into the NdeI/NheI cut pET28a (+) vector in frame. The constructs were transformed into *E. coli* strain BL21(DE3). The *in vitro* phosphorylation assay was performed as previously described [12].

### Immunoblot analysis and *in vivo* phosphorylation assay

Protein extraction was performed as previously described with modification [9]. Open flowers or closed buds at similar stages were collected from 20 inflorescences. The flowers/buds were ground in liquid nitrogen and extracted in 100 µl 1.5X SDS loading buffer. A 15 µl sample was loaded to each lane. The numbers of developing/mature pollen grains should be similar among each sample. However, due to the size difference of the flowers/buds at different developmental stages, different amounts of total proteins were present, which is reflected by the different amount of Rubisco large subunit protein in the Coomassie-blue stained control gels. In this experiment, a comparison of WRKY34 protein levels in an equal number of developing/mature pollen grains is better than in an equal amount of total proteins. A Phos-tag reagent (NARD Institute) was used for the phospho-protein mobility shift assay to detect *in vivo* phosphorylated WRKY34 protein as previously described [9].

### Phylogenetic analysis

The multiple sequence alignment of full-length protein sequences was performed using the ClustalW tool online (http://www.ch.embnet.org/software/ClustalW.html). Phylogenetic trees were constructed and tested by MEGA5 based on the neighbor-joining method [50].

### Quantitative RT-PCR analysis

Total RNA was extracted from each tissue using RNAqueous (Ambion Inc.) according to the manufacturer's instructions. After DNase treatment, µg of total RNA was reverse transcribed, and quantitative PCR analysis was performed using an Optican 2 real-time PCR machine (Bio-Rad). Relative levels of each transcript were calculated after being normalized to the *UBC21* or *EF1α* control.

### DNA-protein electrophoresis mobility shift assay (EMSA)

EMSA was performed as previously described [9]. A synthetic DNA oligonucleotide (5′-CGTTGACCGTTGACCGAGTTGACTTTTTA-3′ with three W boxes underlined) was used as a probe. Two complementary strands of the oligonucleotides were annealed and then labeled at the 5′ end using a T4 polynucleotide kinase. Freshly prepared recombinant WRKY34^WT^ or WRKY34^SA^ protein (1 µg) was incubated with 20,000–50,000 cpm of DNA probe (2 pmole) for 30 min at room temperature in a binding buffer (20 mM HEPES, pH 7.9, 0.1 µg/µL herring sperm DNA, 0.5 mM DTT, 0.1 mM EDTA, 50 mM KCl) in the presence or absence of an unlabeled competitor DNA. The resulting protein-DNA complexes were resolved in 5% non-denaturing polyacrylamide gel in half-strength TBE buffer. Following electrophoresis, the gel was dried onto 3 MM paper and exposed to X-ray film.

## Supporting Information

Figure S1 Expression of *P_LAT52_:4myc-WRKY34* transgene during pollen development. The expression of the *4myc-WRKY34* transgene during pollen development in flowers/buds at different stages was determined by semi-quantitative RT-PCR analysis (upper panel). Primer pair specific to the *4myc-WRKY34* chimeric cDNA was used for PCR. The expression of *UBC21* was used as a reference (lower panel).
(TIF)

Figure S2 Phylogenetic tree of Group I WRKY transcription factors. Unrooted phylogenetic tree of Group I WRKY transcription factors in *Arabidopsis*. Amino acid sequences of Group I WRKY proteins were analyzed by the neighbor-joining method with genetic distance calculated by MEGA5. The numbers at the nodes represent percentage bootstrap values based on 1,000 replications. The length of the branches is proportional to the expected numbers of amino acid substitutions per site, with a scale provided at the bottom of the tree.
(TIF)

Figure S3 FDA viability staining of developing pollen. Developing buds of wild-type (Col-0) and *wrky2-1 wrky34-1* plants were carefully dissected and stained with FDA for pollen viability. Double mutant pollen showed a similar viable rate with wild type in -7 buds (BCP), while the viable rate started to decrease from -6 buds (BCP) in comparison with the wild type. For each stage, 50–100 pollen grains were counted. Presented result is from two repeats. Error bar  =  standard error.
(TIF)

Figure S4 TEM image of aborted *wrky2-1 wrky34-1* pollen. Bar = 2 µm.
(TIF)

Figure S5 Comparable levels of *WRKY34* expression in pollen grains from wild-type (Col-0) and *wrky2-1 wrky34-1* double mutant complemented with wild-type *WRKY34^WT^* or loss-of-phosphorylation *WRKY34^SA^*. Quantitative RT-PCR of *WRKY34* expression in wild type (Col-0), *wrky2-1 wrky34-1 P_WRKY34_:WRKY34^WT^*, and *wrky2-1 wrky34-1 P_WRKY34_:WRKY34^SA^* plants. Error bar = standard derivation.
(TIF)

Figure S6 Mutation of six Ser residues to Ala in WRKY34 does not alter its W-box binding activity. Electrophoretic mobility shift assay (EMSA) was performed using freshly prepared recombinant WRKY34^WT^ or WRKY34^SA^ protein and ^32^P-labeled W-box probe. The specificity of W-box binding activity was demonstrated by competition assay using 250-fold excess unlabeled W-box, GCC-box, or AS1-box DNAs.
(TIF)
