# Supplementary figures and images for: Phosphorylation of a WRKY Transcription Factor by MAPKs Is Required for Pollen Development and Function in Arabidopsis
Source: PLoS Genet. 2014 May 15;10(5):e1004384. doi: 10.1371/journal.pgen.1004384 (PMC4022456; doi:10.1371/journal.pgen.1004384)

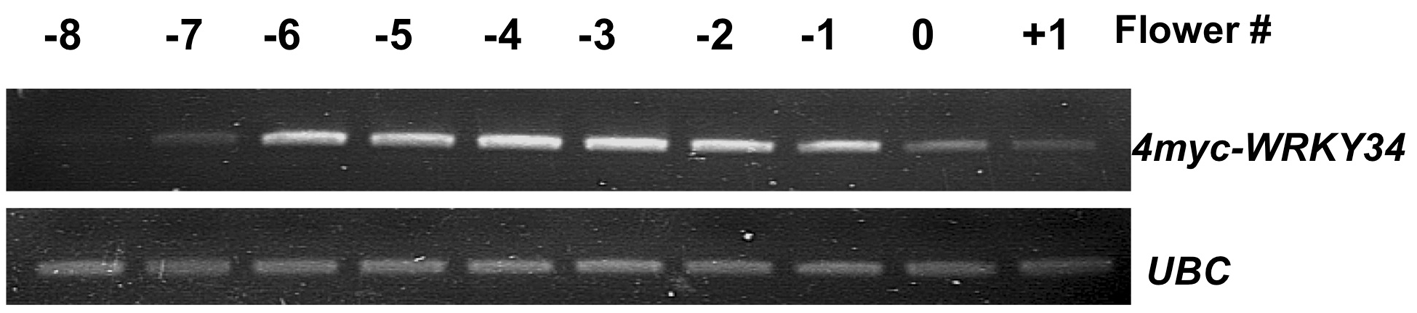

Supplement: Figure S1 — Expression of PLAT52:4myc-WRKY34 transgene during pollen development. The expression of the 4myc-WRKY34 transgene during pollen development in flowers/buds at different stages was determined by semi-quantitative RT-PCR analysis (upper panel). Primer pair specific to the 4myc-WRKY34 chimeric cDNA was used for PCR. The expression of UBC21 was used as a reference (lower panel). (TIF) [file pgen.1004384.s001.tif]

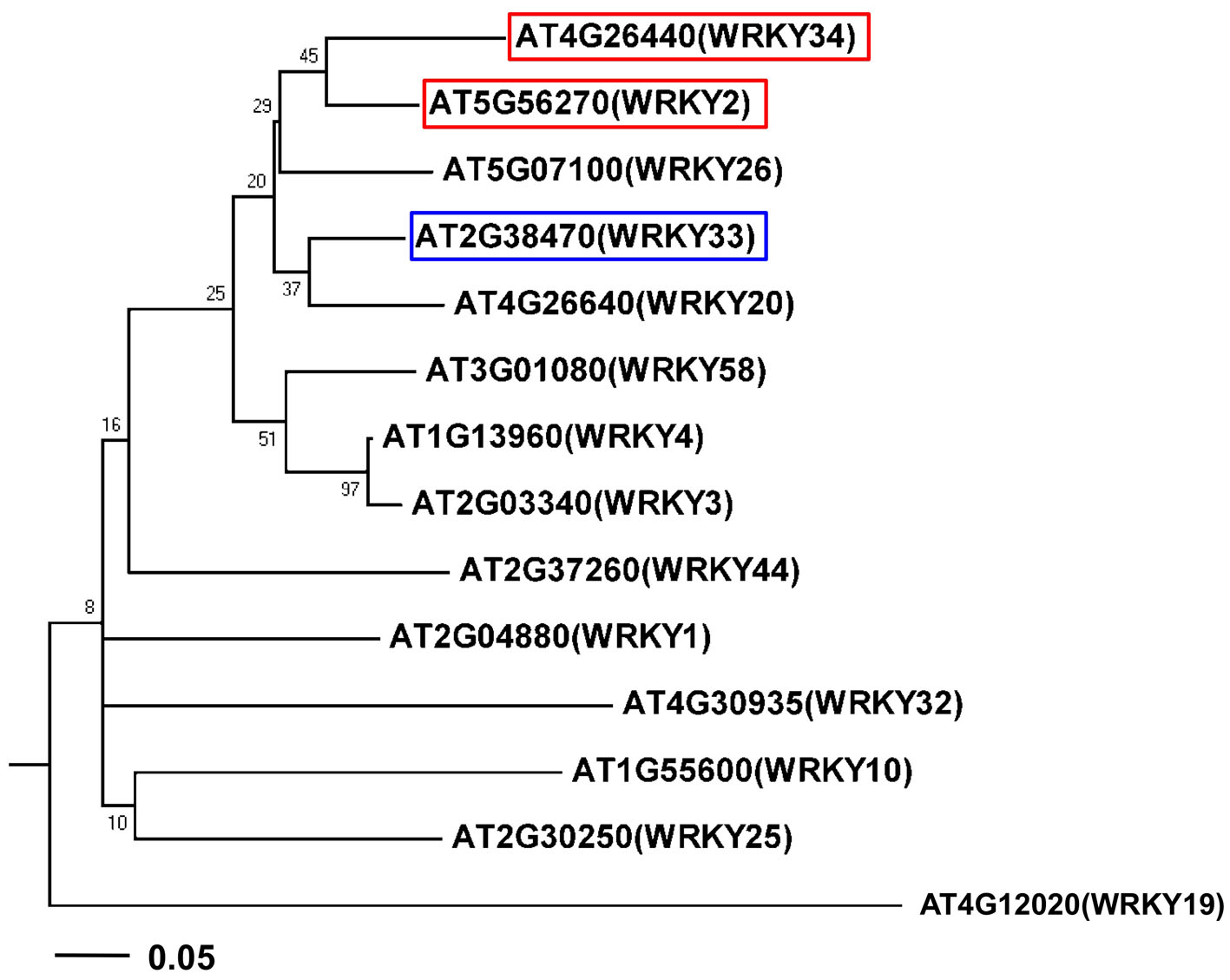

Supplement: Figure S2 — Phylogenetic tree of Group I WRKY transcription factors. Unrooted phylogenetic tree of Group I WRKY transcription factors in Arabidopsis. Amino acid sequences of Group I WRKY proteins were analyzed by the neighbor-joining method with genetic distance calculated by MEGA5. The numbers at the nodes represent percentage bootstrap values based on 1,000 replications. The length of the branches is proportional to the expected numbers of amino acid substitutions per site, with a scale provided at the bottom of the tree. (TIF) [file pgen.1004384.s002.tif]

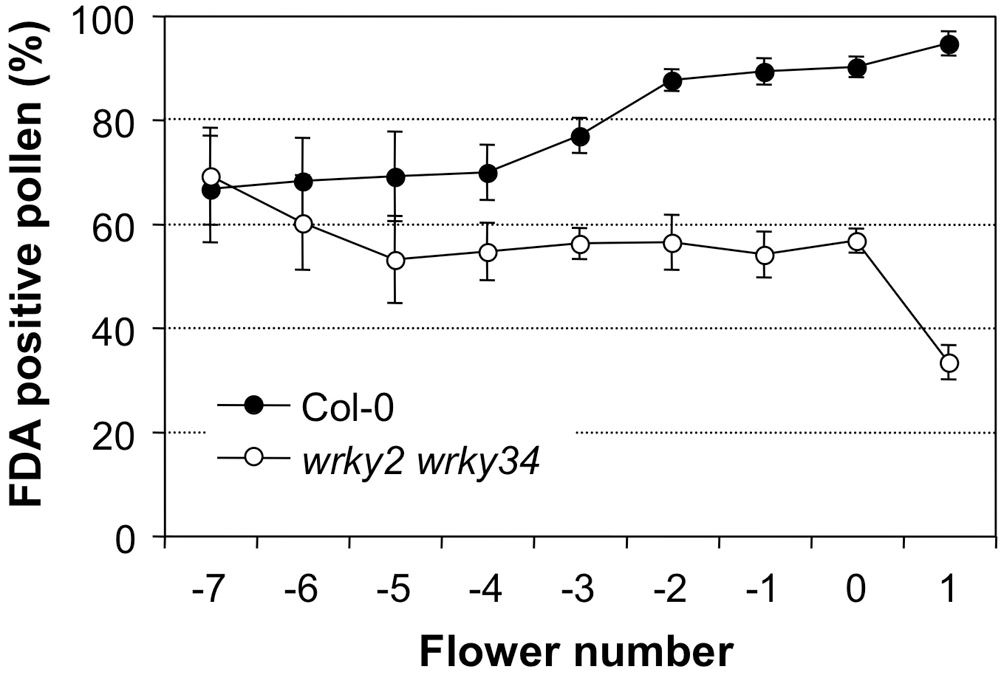

Supplement: Figure S3 — FDA viability staining of developing pollen. Developing buds of wild-type (Col-0) and wrky2-1 wrky34-1 plants were carefully dissected and stained with FDA for pollen viability. Double mutant pollen showed a similar viable rate with wild type in -7 buds (BCP), while the viable rate started to decrease from -6 buds (BCP) in comparison with the wild type. For each stage, 50–100 pollen grains were counted. Presented result is from two repeats. Error bar = standard error. (TIF) [file pgen.1004384.s003.tif]

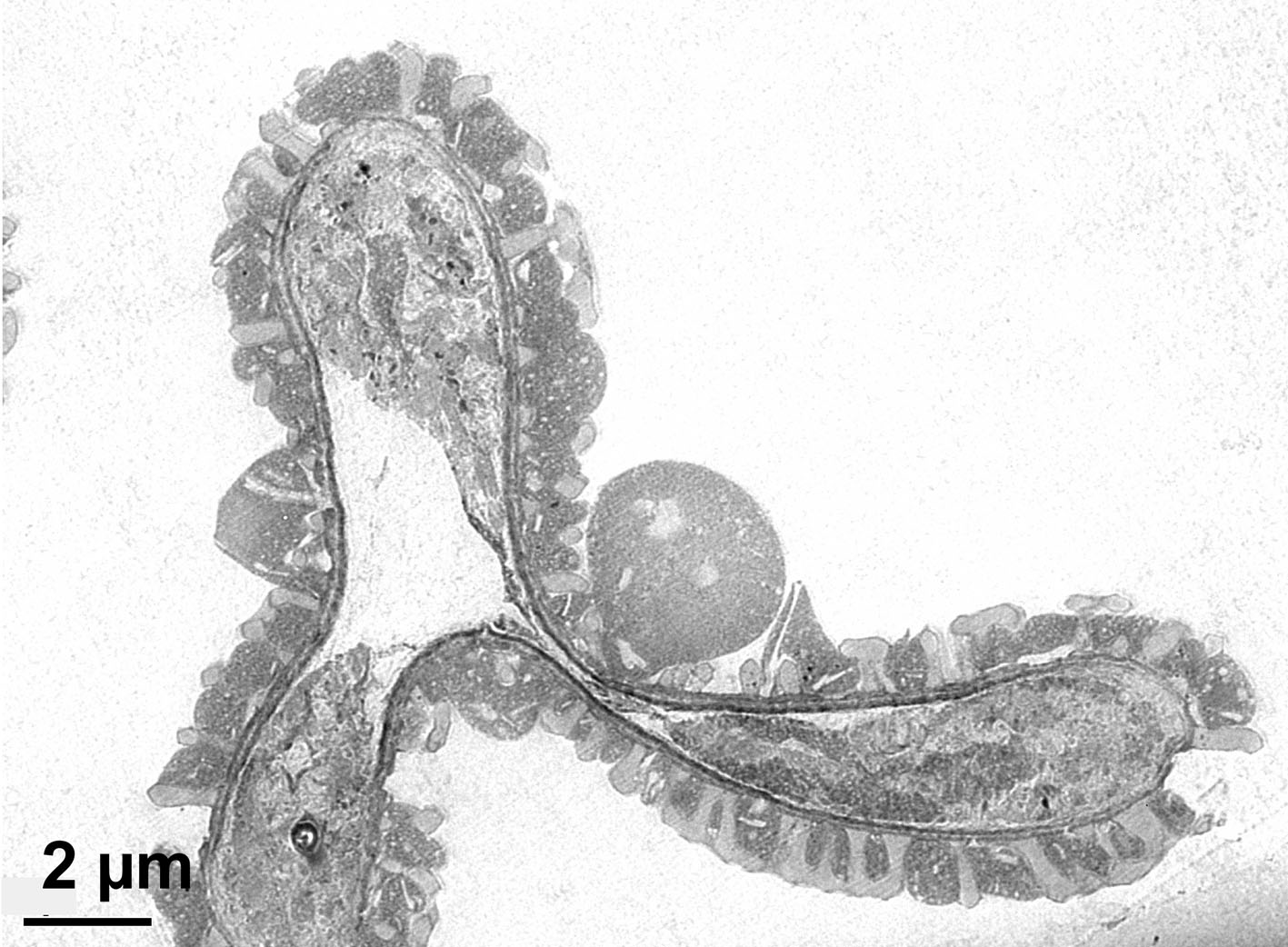

Supplement: Figure S4 — TEM image of aborted wrky2-1 wrky34-1 pollen. Bar = 2 µm. (TIF) [file pgen.1004384.s004.tif]

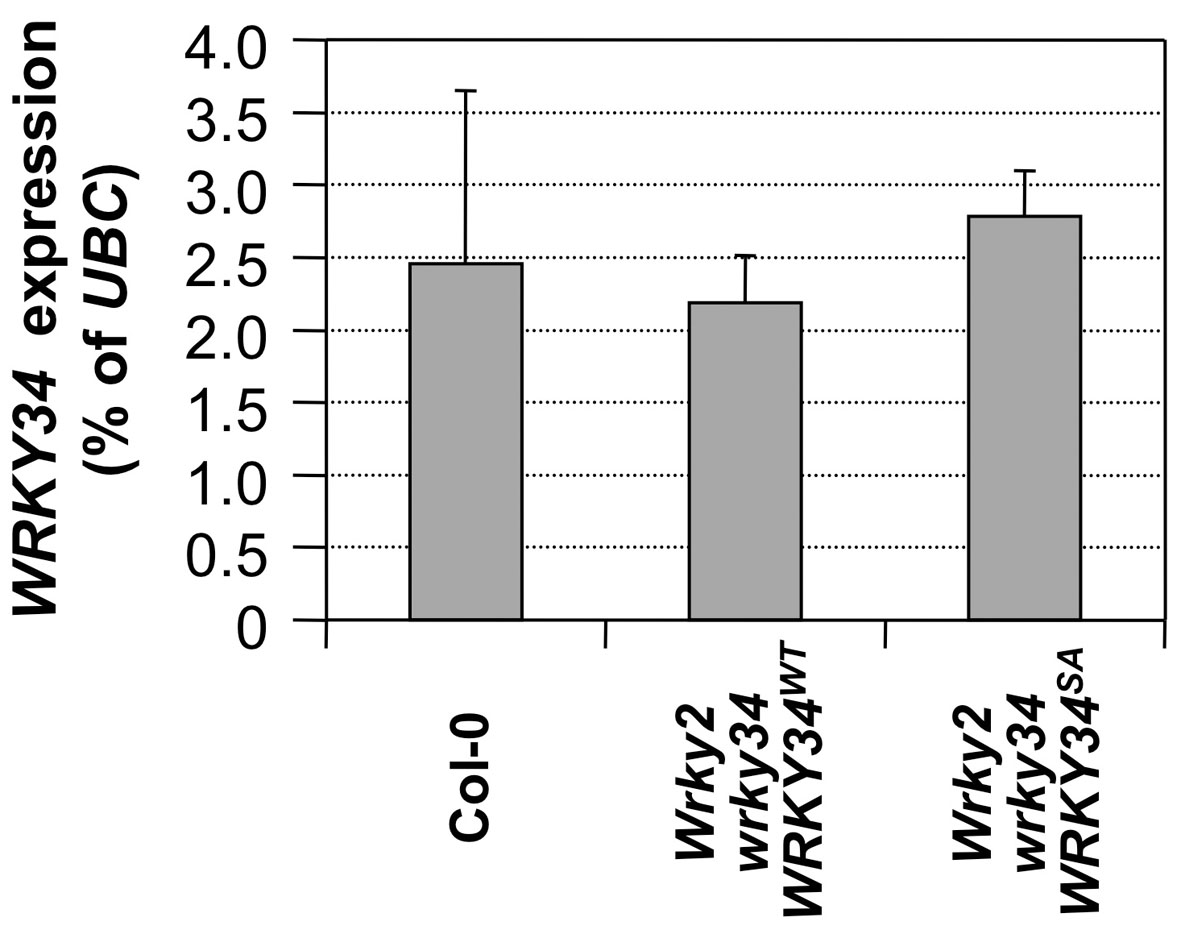

Supplement: Figure S5 — Comparable levels of WRKY34 expression in pollen grains from wild-type (Col-0) and wrky2-1 wrky34-1 double mutant complemented with wild-type WRKY34WT or loss-of-phosphorylation WRKY34SA. Quantitative RT-PCR of WRKY34 expression in wild type (Col-0), wrky2-1 wrky34-1 PWRKY34:WRKY34WT, and wrky2-1 wrky34-1 PWRKY34:WRKY34SA plants. Error bar = standard derivation. (TIF) [file pgen.1004384.s005.tif]

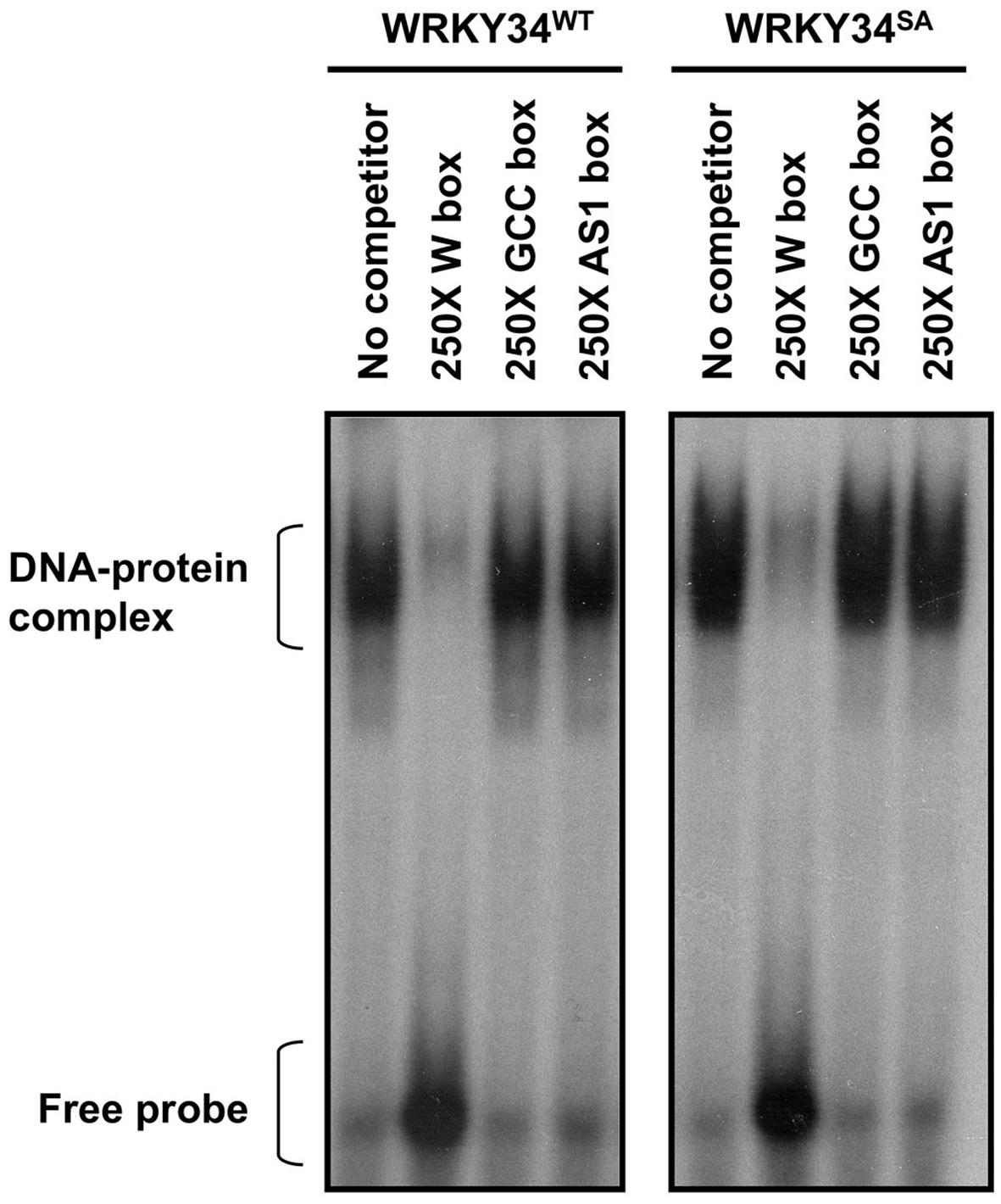

Supplement: Figure S6 — Mutation of six Ser residues to Ala in WRKY34 does not alter its W-box binding activity. Electrophoretic mobility shift assay (EMSA) was performed using freshly prepared recombinant WRKY34WT or WRKY34SA protein and 32P-labeled W-box probe. The specificity of W-box binding activity was demonstrated by competition assay using 250-fold excess unlabeled W-box, GCC-box, or AS1-box DNAs. (TIF) [file pgen.1004384.s006.tif]
